# Plant regeneration from seeds responds to phylogenetic relatedness and local adaptation in Mediterranean *Romulea* (Iridaceae) species

**DOI:** 10.1002/ece3.2150

**Published:** 2016-05-24

**Authors:** Angelino Carta, Sarah Hanson, Jonas V. Müller

**Affiliations:** ^1^ Department of BiologyUniversity of Pisavia Derna 1I‐56126PisaItaly; ^2^ Royal Botanic Gardens, Kew, Millennium Seed Bank, Conservation ScienceWakehurst PlaceArdinglyWest SussexRH17 6TNUK

**Keywords:** annual grasslands, embryo growth, evolution, life cycle, niche differentiation, seed dormancy, species divergence, temporary wetlands

## Abstract

Seed germination is the most important transitional event between early stages in the life cycle of spermatophytes and understanding it is crucial to understand plant adaptation and evolution. However, so far seed germination of phylogenetically closely related species has been poorly investigated. To test the hypothises that phylogenetically related plant species have similar seed ecophysiological traits thereby reflecting certain habitat conditions as a result of local adaptation**,** we studied seed dormancy and germination in seven Mediterranean species in the genus *Romulea* (Iridaceae). Both the across‐species model and the model accounting for shared evolutionary history showed that cool temperatures (≤ 15°C) were the main factor that promoted seed germination. The absence of embryo growth before radicle emergence is consistent with a prompt germination response at cool temperatures. The range of temperature conditions for germination became wider after a period of warm stratification, denoting a weak primary dormancy. Altogether these results indicate that the studied species exhibit a Mediterranean germination syndrome, but with species‐specific germination requirements clustered in a way that follows the phylogenetic relatedness among those species. In addition, species with heavier seeds from humid habitats showed a wider range of conditions for germination at dispersal time than species from dry habitats possessing lighter seeds. We conclude that while phylogenetically related species showed very similar germination requirements, there are subtle ecologically meaningful differences, confirming the onset of adaptation to local ecological factors mediated by species relatedness.

## Introduction

Knowledge of plant evolution and species delimitation may be limited by a lack of information on the reproductive biology of plant species and how it fits into their life cycles (Grant 1981; De Queiroz 2007; Stuessy 2009). Sexual reproduction and seed germination are two of the most important events in the life cycle of flowering plants. While sexual reproduction involves pollen transfer from anther to stigma, thereby enabling fertilization and seed development (Barrett 2002), seed germination is completed when the embryonic root visibly emerges through the outer structures of the seed (radical emergence; Bewley et al. 2013). The timing of germination is affected by the environmental conditions which prevail during seed maturation and after seed dispersal. The timing of germination also affects the probability of a seedling to survive (Donohue et al. 2010; Baskin and Baskin 2014). Germination is influenced by a large number of genes and environmental factors and is often mediated by seed dormancy (Koornneef et al. 2002; Finch‐Savage and Leubner‐Metzger 2006; Bewley et al. 2013). If a seed is dormant, then certain environmental conditions are required to break its dormancy, while other environmental factors promote germination of seeds that have become nondormant. By definition, a seed which is able to germinate over the widest possible range of conditions is nondormant, while any narrowing of this range indicates an increase in the degree of dormancy. Seed dormancy is therefore a quantitative trait of great adaptive importance, which evolved to cue emergence of seedlings at the environmentally most advantageous time and place (assuming that seed dispersal itself is not a limiting factor; Vleeshouwers et al. 1995; Fenner and Thompson 2005; Willis et al. 2014).

Physiological processes that regulate seed dormancy and germination often differ among species and may depend on their phylogeny, geographic distribution, habitat, and life cycle (Grime et al. 1981; Schütz and Rave 1999; Thompson and Ceriani 2003; Vandelook et al. 2008). However, Baskin and Baskin (2014) reported contradicting evidence for the relationship between seed dormancy and germination on the one and habitat and life history on the other side; these correlations should be interpreted carefully (Schütz and Rave 1999). Nevertheless, bearing in mind that physiological properties and dormancy patterns are related to the phylogenetic position of the species (Nikolaeva 1999), closely related species with similar geographic distributions and life cycles should be considered in order to study the correlation between different germination requirements among species and distinct habitats (Baskin and Baskin 2014). In this context, when performing statistical analyses, one must take the fact into account that closely related species tend to be more similar than distantly related ones, in other words that species cannot be considered as statistically independent units of observation (Pagel 1999). This approach of relating germination strategies to habitat conditions at species level helps explain reproductive biology of plant species and may give an insight into their evolutionary history.

We applied this approach to seven Mediterranean species of the genus *Romulea* Maratti (Asparagales, Iridaceae). This genus is comprised of about 100 taxa and displays a disjunct distribution pattern, with about 80% of the taxa occurring in southern Africa and on the Arabian Peninsula and the other 20% (about 15 species) occurring in the western parts of the Mediterranean Basin (Goldblatt et al. 2008), where they are found in coastal and island ecosystems (Frignani and Iiriti 2011). While extensive morphological, anatomical, and taxonomical studies on Mediterranean *Romulea* species already exist (Işik and Dönmez 2007; Coppi et al. 2010; Frignani and Iiriti 2011; Peruzzi et al. 2011; Karaismailoglu 2015; Mifsud 2015), relationships among individual taxa have not been investigated sufficiently, and our knowledge on their ecology, physiology, and evolutionary history is limited (Kök et al. 2007; Angiolini et al. 2015). Mediterranean *Romulea* species typically grow in dry or temporarily humid grasslands and pastures mainly comprised of annual species (Čarni et al. 2014). Such habitats are highly variable in space and time, and they are characterized by high levels of biodiversity under intermediate intensities of environmental disturbance (Grime 2006). Frignani and Iiriti (2011) pointed to a high degree of polymorphism and formulated the hypothesis that adaptation to local ecological conditions should be considered among the main evolutionary drivers of inter‐ and intraspecific variability within Mediterranean taxa, for one of which, *Romulea bulbocodium* (L.) Sebast. & Mauri, two ecotypes were recently identified (Angiolini et al. 2015). In this article, we analyze this question studying the functional ecology of *Romulea* species and their association with environmental conditions, something which has never been analyzed before. We test the hypothesis that phylogenetically closely related species show differences in ecophysiological traits such as dormancy and germination characteristics, thereby reflecting the distinct habitats and bioclimatic areas in which they occur. Using outdoor and laboratory experiments, we investigated (i) the extent to which embryos grow before radicle emergence occurs along with the phenology of radicle and shoot emergence and (ii) the influence of warm stratification and of light and temperature on radicle emergence. We applied across‐species models and phylogenetic regressions to examine the driving factors for the evolution of seed traits and regeneration strategies. Specifically, our aim was to verify to what extent seed germination has evolved in relation to local ecological conditions by investigating whether species respond selectively to distinct seed germination cues, as a means of species habitat preference.

## Materials and methods

### Seed material

We studied seven species of the genus *Romulea* (Table 1), all of which are early spring‐flowering geophytes that develop leaves and flowers from a subterraneous perennial corm. These species were selected to represent a very wide geographic and ecological spectrum present within the genus *Romulea* in the Mediterranean Basin. *R. columnae* and *R. ramiflora* are widely distributed in the Mediterranean Basin. *R. ligustica*,* R. variicolor,* and *R. requienii* are central Mediterranean endemics (Frignani and Iiriti 2011; Mifsud 2015), while *R. insularis* and *R. linaresii* are narrow endemics occurring on Capraia Island (Tuscan Archipelago) and northwest Sicily, respectively (Frignani and Iiriti 2011; Foggi et al. 2015). Table 1 shows the different ecological conditions and habitat preferences of the species.

**Table 1:** List of studied *Romulea* species, populations collected, habitat descriptions, and bioclimatic variables of the collection sites

Species	Collecting site									Seed properties	
	Locality	Lat y	Long x	Habitat	Altitude (m)	T	Ts	Pw	Ps	Mass (g)	Length (mm)
*R. columnae* Sebast. & Mauri	Italy, Tuscany, Vecchiano, Sassi Grossi	43.809	10.39478	arid oligotrophic grasslands	30	14.4	21.9	243	123	0.26 ± 0.01b	1.38 ± 0.01de
	Italy, Tuscany: Montecatini Val di Cecina, Casaglia	43.349	10.66327	arid oligotrophic grasslands	220	14.2	21.8	219	117	0.25 ± 0.01b	1.49 ± 0.02f
	Italy, Tuscany: Isola d'Elba, Pietra Murata	42.757	10.18183	arid oligotrophic grasslands	550	15.4	22.4	176	94	0.24 ± 0.01b	1.31 ± 0.01 cd
	France, Corsica: Calcatoggio, Pevani	42.018	8.6999	arid oligotrophic grasslands	240	14.1	20.8	230	85	0.18 ± 0.01a	1.30 ± 0.01c
	Malta: Wied Incita, Attard	35.894	14.43655	arid oligotrophic grasslands	80	18.4	25.1	174	42	0.25 ± 0.01b	1.32 ± 0.02 cd
*R. insularis* Sommier	Italy, Tuscany: Isola di Capraia, Sella dell'Acciatore	43.032	9.80718	temporary wetlands	250	14.9	21.9	219	111	0.17 ± 0.01a	1.08 ± 0.01a
*R. ligustica* Parl.	Italy, Sardinia: Olmedo, near Lago del Cuga	40.619	8.46662	arid mesotrophic grasslands	100	15.8	22.7	237	54	0.38 ± 0.03 cd	1.29 ± 0.01c
	Italy, Sardinia: Sinnai, Maracalagonis, Monte Cresia	39.303	9.39148	arid mesotrophic grasslands	400	15.1	22.4	163	56	0.35 ± 0.01c	1.38 ± 0.02de
*R. linaresii* Parl.	Italy, Siciliy: Trapani, foothills of Monte Cofano	38.098	12.65905	wet clayey sligthly salty grasslands	10	17.4	24.1	156	53	0.47 ± 0.01 fg	1.88 ± 0.02i
*R. variicolor* Misfud	Italy, Sicily: Ragusa, Scicli	36.722	14.69181	wet clayey sligthly salty grasslands	30	16.8	23.7	144	40	0.41 ± 0.01de	1.45 ± 0.01ef
	Malta: Gozo, Gharb	36.064	14.20615	wet clayey sligthly salty grasslands	80	18.2	24.8	164	40	0.41 ± 0.01de	1.71 ± 0.01 h
*R. ramiflora* Ten.	Italy, Tuscany: Isola d'Elba, Lacona	42.761	10.30709	wet clayeysligthly salty grasslands	4	15.4	22.4	176	94	0.43 ± 0.03def	1.68 ± 0.01 h
	France, Corsica: Farinole, Grotta u Banditu	42.725	9.33534	arid mesotrophic grasslands	20	13.4	20.3	239	109	0.49 ± 0.01gh	1.74 ± 0.01 h
	Italy, Sicily: Ragusa, Marina di Modica	36.719	14.74349	arid mesotrophic grasslands	5	16.8	23.7	144	40	0.46 ± 0.01eg	1.58 ± 0.01 g
*R. requienii* Parl.	France, Corsica: Ajaccio, Capo di Feno	41.911	8.64311	temporary wetlands	10	14.7	21.4	219	77	0.16 ± 0.02a	1.20 ± 0.01b
	Italy, Sardinia: Olbia, Coda Cavallo	40.832	9.70729	temporary wetlands	10	16.1	22.9	189	55	0.28 ± 0.01b	1.48 ± 0.02f
	Italy, Sardinia: Gesturi, Giara	39.740	8.98858	temporary wetlands	580	15.2	22.6	231	58	0.25 ± 0.01b	1.28 ± 0.02c

T = mean annual temperature (°C); Ts = mean summer temperature (°C); Pw = winter precipitation (mm); Ps = summer precipitation (mm). Average seed mass (mean ± SE of five replicates of 100 seeds each) and seed length (mean ± SE of 30 seeds). Means with different letters are significantly different from each other (*P *<* *0.05, Tukey's multiple comparisons test).

For each species, multiple populations were sampled, with the exception of *R. insularis* and *R. linaresii* because of their narrow distribution. Seed capsules (fruits) were collected at the time of natural dispersal in May–June 2014 from about 300 plants for each population. Ripe fruits were then spread out in the laboratory to allow them to open and the seeds to fall out. To minimize potentially damaging and/or dormancy breaking effects of after‐ripening, the experiments were started within 1 month of fruit collection.

Seed mass was determined by weighing five replicates of 100 seeds each per accession (seeds equilibrated at approximately 20°C and 50% RH). We also measured the maximum length of 30 seeds using a digital caliper for each species.

### Phenology of radicle and seedling emergence outdoors

The aim of these experiments was to describe the phenology of radicle and seedling emergence using seeds kept under quasi‐natural conditions. Seeds were sown in pots (40 × 20 × 20 cm) filled with loam and sand (2:1 v/v) at Pisa Botanical Garden on 1 July 2014. Pots were placed outdoors in the garden, and the air temperature was recorded throughout the duration of the experiment. Pots were watered to field capacity four times a month throughout the year to maintain field capacity, with the exception of July and August, when they were watered only twice a month to simulate the summer drought common in the Mediterranean Basin (Copete et al. 2011; Carta et al. 2014).

To study the phenology of radicle emergence, for each population, 20 groups of 50 seeds each were mixed with fine‐grained sand. Each group was placed in a fine‐mesh polyester bag, labeled, and buried 5 cm deep in the pots. To study the phenology of shoot emergence, for each population, 1 pot was filled with the growing medium and 100 seeds were sown equidistant from each other and buried at a depth of 0.5 cm.

Every 10 days, a bag was exhumed and germinated seeds were counted. Observations of cotyledon emergence were made every 10 days until the end of the experiments.

### Embryo growth and radicle emergence in the laboratory

Experiments were conducted in temperature and light controlled conditions using a 12 h daily thermo‐ (for alternating temperatures) and photo‐period (= light hereafter). Light was provided during the warm phase for the alternating temperature regimes by white fluorescent tubes (40–50 *μ*mol m^−2^s^−1^).

For all germination experiments, we used 90‐mm‐diameter Petri dishes containing 1% distilled water agar, with three samples of 20 seeds for each population and observations made every seven days. A move‐along experiment (*sensu* Baskin and Baskin 2003) was conducted to study germination phenology in the laboratory. Seeds were exposed to temperature changes simulating the natural seasonal summer → autumn → winter sequence in the sampling areas. This experiment was conducted under both light and complete dark conditions. Continuous darkness was achieved by wrapping Petri dishes in aluminium foil for the duration of the experiment.

Summer conditions were defined as 60 days at 25/15°C (20°C for the constant temperature regime), autumn conditions as 30 days at 20/10°C (15°C for the constant temperature regime), and winter conditions as 60 days at 15/5°C (10°C for the constant temperature regime). The constant temperature regime was chosen in order to simulate natural conditions with low levels of disturbance, such as under a closed canopy or in the soil.

To study the effect of mean temperature, temperature regime and light on radicle emergence, in addition to the experiments described above, seeds were directly exposed to diurnal alternating (15/5, 20/10, and 25/15°C) or constant temperatures (10, 15, and 20°C) without any pretreatment. These experiments lasted for 50 days and were conducted under both light and complete dark conditions. The number of seeds and samples for each test and observation was as described above.

To study whether the species have an underdeveloped embryo at the moment of natural dispersal, we also studied whether the embryo had to grow within the seed before germination can occur. At the beginning of the experiments (T_0_), 15 seeds of each population were imbibed in distilled water and then dissected, and their embryo lengths measured under a microscope with a micrometer. The ratio of embryo length to seed length (E:S) was then calculated. Then, for each population, two other groups of 15 seeds each were placed on agar and then kept at a constant temperature of 10°C (considered optimal, see below). To get measurements of embryo growth, seeds were dissected after 10 (T_1_) and after 20 days (T_2_). We decided to conclude this experiment after 20 days because soon after this time, seeds start to germinate. The E:S ratio of seeds that had germinated was not determined; instead, for these seeds, the critical E:S ratio was used. The critical E:S ratio is defined as the average E:S ratio of 20 randomly selected seeds with split seed coat, but no radicle protrusion.

### Effect of summer condition on radicle emergence

To examine whether warm stratification is necessary to promote germination or to break dormancy, seeds were placed directly into autumn (20/10°C alternating or 15°C constant temperatures) and then into winter conditions (15/5°C alternating or 10°C constant temperatures), in both light and dark. The number of seeds and samples for each test and observation was as described above.

### Statistical analysis

All calculations were performed based on viable seed only and analyzed using the R environment for statistical computing (R Development Core Team 2015).

Seed morphological traits (mass and length) and embryo growth data were analyzed by means of analysis of variance (ANOVA). Embryo growth data were described using linear regressions.

For each species, the seed germination responses were analyzed by fitting factorial generalized linear mixed models (GLMMs, logit link function, binomial distribution) to the germination response data using the R package lm4 (Bates et al. 2014) with incubation conditions (mean temperature, temperature regimes, and light) and pretreatments (warm stratification) as fixed predictors and considered population as a random effect (Bolker et al. 2009). For species with a single population, generalized linear models (GLMs) with a logit link function and a binomial error structure were fitted.

To describe the germination process, the Weibull function was fitted to cumulative germination data using the drc package (Ritz and Streibig 2005). Goodness of regression was assessed by graphical analysis of residuals and *F*‐test sums of squares for lack of fit. We used *F*‐test sums of squares to assess whether individual germination curves could be constrained to a single regression. The effect of different treatments on the germination rate was also compared considering the time to reach 50% germination (= t_50_) extracted from the fitted values of the curves.

To examine whether seed germination rates (t_50_) were influenced by seed mass and phylogenetic relationships, simple phylogenetic generalized least‐squares (pgls) were fitted by maximum likelihood in the ape and caper package (Paradis et al. 2004; Orme et al. 2012), considering each population as a separate observation unit. Pagel's *λ* was used as a measure of the phylogenetic signal (Pagel 1999), and this is the extent to which correlations in traits reflect their shared evolutionary history. The phylogenetic framework for *Romulea* used in this study was derived from the work of Coppi et al. (2010), who constructed a molecular phylogeny. *Crocus* was considered as an out‐group (Harpke et al. 2015), for which three species with seed germination data were used (Carta et al. 2014). To date nodes in the tree, we considered the divergence age estimate *Romulea* vs. *Crocus*, 12.5 Mya, taken from Chen et al. (2013), and scaled phylogenetic branch lengths using the Bladj algorithm in Phylocom (Webb et al. 2009). Finally, to summarize the seed germination response, we used a generalized estimating equation (GEE) procedure using the compar.gee function within the ape package (Paradis and Claude 2002). This procedure uses a GLM approach (logit link function, binomial distribution) incorporating the phylogenetic relatedness among species as a correlation matrix in the model (phylogenetic logistic model).

We also analyzed the seed germination using GLM across‐species and embryo growth data using standard regressions without accounting for phylogeny in order to compare the results with the model that controlled for shared evolutionary history.

To determine whether the germination response could be predicted from the local environment of the populations, we fitted a GLM to germination results using the local values of bioclimatic variables as predictors: mean annual temperature, mean summer temperature, winter precipitation, and summer precipitation (Table 1). We acquired climatic data of the sampling sites from the WorldClim website (http://www.worldclim.org, Hijmans et al. 2005). In addition, habitat moisture, warm stratification, and seed mass were considered in the model. The species were classified into three categories according to habitat moisture (temporarily wet = 3; moist = 2; dry = 1). For each analysis, a full model including all main factors and interactions was fitted. The full model was then reduced by testing all possible combinations of variables, in order to retain the main explanatory variables and the model with the lowest Akaike's information criterion (AIC).

## Results

### Phenology of radicle and seedling emergence outdoors

**Figure 1:**
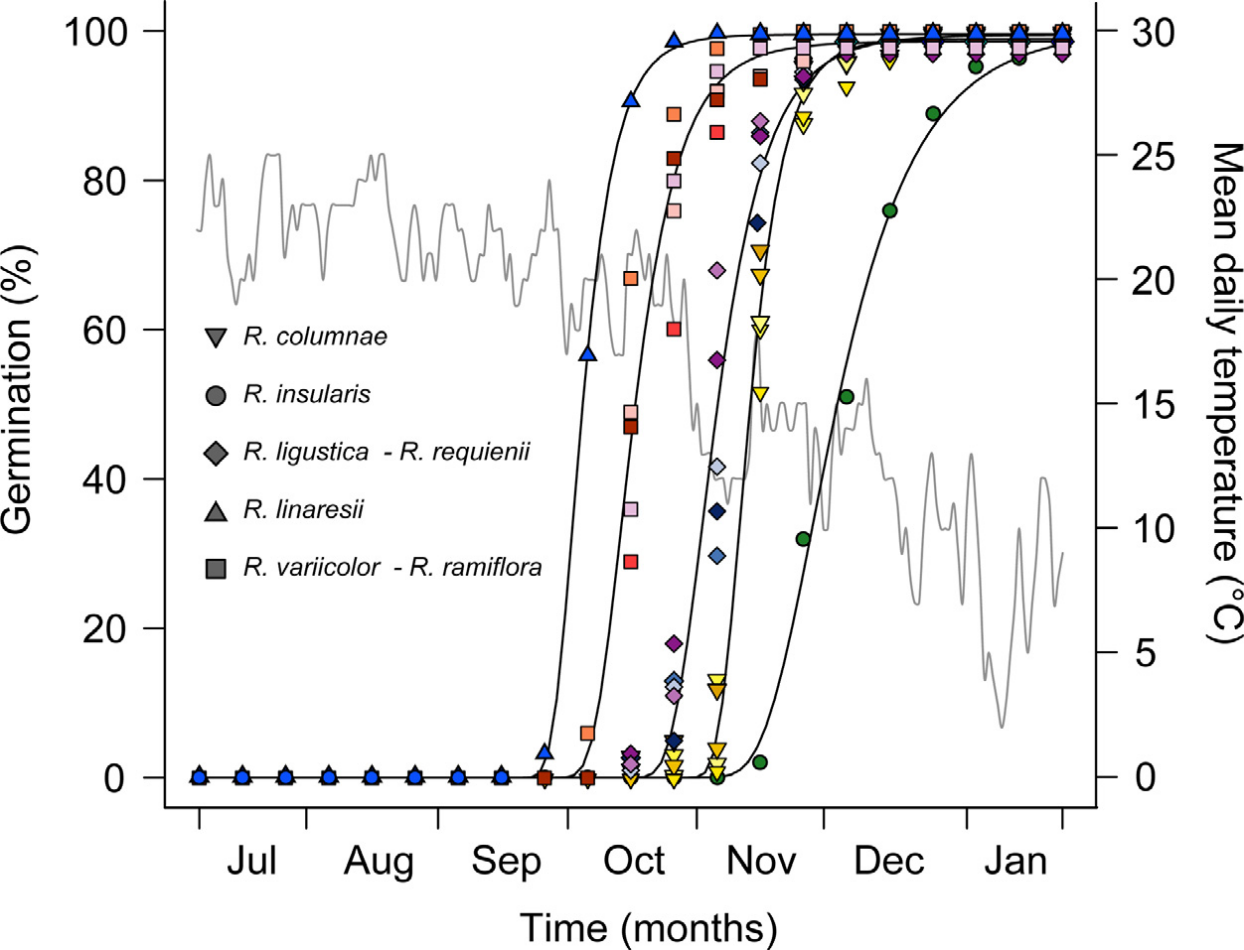
Radicle emergence of *Romulea* species in a common garden. The data for radicle emergence for seeds that could be constrained to single lines without a significant increase in residual deviance (*P *>* *0.05) are represented by the same symbols (as indicated) with different colours for each population and superimposed onto cumulative radicle emergence percentage curves fitted using the Weibull function. The grey line indicates the mean daily temperature.

The phenology of radicle emergence did not vary among the populations of any particular species. Instead, the radicle emergence varied significantly (*P *<* *0.001) among clusters of species, with *R. linaresii* showing the most rapid emergence (September) followed by the cluster *R. variicolor *+ *R. ramiflora* (Fig. 1). The *R. ligustica*+*R. requienii* populations followed about 20 days later. Their radicle progress curve was significantly (*P *<* *0.001) different from that of *R. columnae* populations. The most delayed species was *R. insularis* whose radicle emergence was concluded in January.

Seed mass, which was significantly (*P *<* *0.05) different among the species (Table 1) and positively related to seed length (*r* ^2^ = 0.83, *P *<* *0.001), significantly explained the germination rate (t_50_) in both simple (*r* ^2^ = 0.82, *P *<* *0.001) and phylogenetic regressions (*r* ^2^ = 0.2, *P *<* *0.05). Phylogenetic regression of germination rates (t_50_) also revealed that *λ* did not differ significantly from unity (*λ *= 1, CI 0.86, 1), implying that the phenology of seed germination is more similar than expected from the phylogenetic relationships of the species.

### Embryo growth and radicle emergence in the laboratory

**Figure 2:**
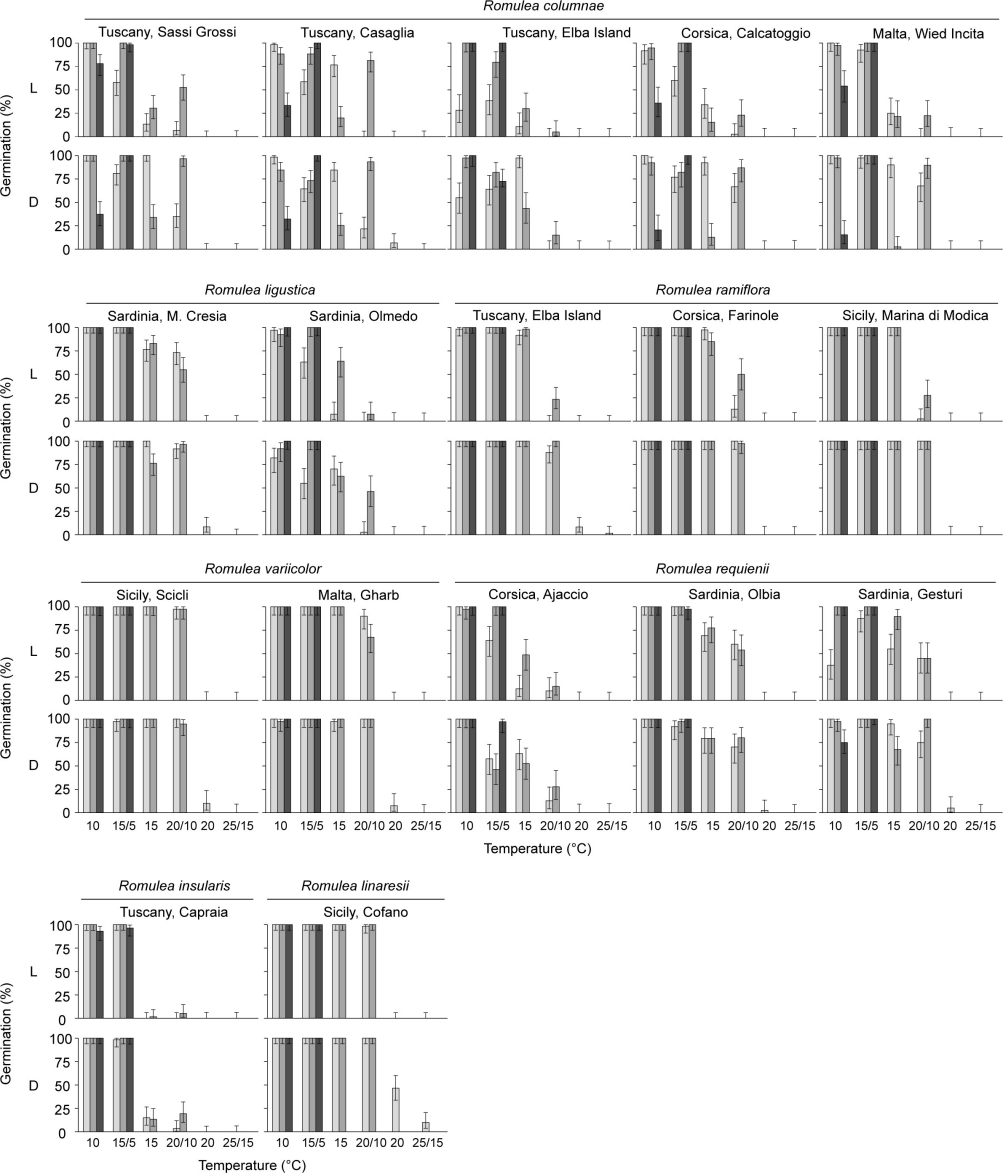
Final radicle emergence percentage (mean ±95% binomial CI) exposed to 12‐h daily light photoperiod (L) or in continuous darkness (D) and incubated at 10, 15/5, 15, 20/15, 20, or 25/15°C. For simulated winter constant temperature (10°C) or alternating temperature regimes (15/5°C), seeds were sown fresh (white columns) or after 30 days of autumn temperatures (15 or 20/10°C, gray columns) or warm stratified at 20°C (or 25/15°C) whereupon they were placed at simulated autumn temperatures (dark gray columns). For simulated autumn constant temperature (15°C) or alternating temperature regimes (20/10°C), seeds were sown fresh (white columns) or after 30 days of warm stratification (20 or 25/15°C, gray columns). For simulated summer constant temperature (20°C) or alternating temperature regimes (25/15°C), seeds were only sown fresh.

Embryos of all populations did not significantly differ at the three observation steps (Table 2). None of the standard regressions showed a significant embryo growth, at the species level or at the whole genus level. However, using a phylogenetic regression, a slightly significant (*P *=* *0.04) embryo growth was detected. For all species, germination was relatively rapid (10–20 days). Radicles did not emerge during the simulated summer conditions, and mean temperature was a significant negative term (*P *<* *0.001) in most models (Table 3, Table 4), indicating that germination is favoured by cool temperatures (≤ 15°C). Indeed, germination started when seeds were moved from summer to autumn conditions or when they were directly placed into autumn or winter conditions (Fig. 2).

**Table 2:** Embryo growth (mean E:S ratio ± SE) in seeds of *Romulea* species after 0 (T0), 10 (T1), and 20 (T2) days under near optimal light and temperature (10°C) conditions for germination

Species	Population	T0 (0 day)	T1 (10 days)	T2 (20 days)
*R. columnae*	Italy, Tuscany, Vecchiano, Sassi Grossi	0.58 ± 0.01a (0%)	0.58 ± 0.01a (0%)	0.59 ± 0.01a (1%)
	Italy, Tuscany: Montecatini Val di Cecina, Casaglia	0.53 ± 0.01a (0%)	0.53 ± 0.01a (0%)	0.52 ± 0.01a (17%)
	Italy, Tuscany: Isola d'Elba, Pietra Murata	0.54 ± 0.01a (0%)	0.55 ± 0.01a (0%)	0.53 ± 0.01a (2.5%)
	France, Corsica: Calcatoggio, Pevani	0.63 ± 0.01a (0%)	0.63 ± 0.02a (0%)	0.65 ± 0.02a (0%)
	Malta: Wied Incita, Attard	0.53 ± 0.01a (0%)	0.51 ± 0.01a (0%)	0.54 ± 0.01a (2.5%)
*R. insularis*	Italy, Tuscany: Isola di Capraia, Sella dell'Acciatore	0.66 ± 0.01a (0%)	0.66 ± 0.01a (0%)	0.67 ± 0.01a (1%)
*R. ligustica*	Italy, Sardinia: Olmedo, near Lago del Cuga	0.63 ± 0.01a (0%)	0.64 ± 0.01a (0%)	0.64 ± 0.01a (2%)
	Italy, Sardinia: Sinnai, Maracalagonis, Monte Cresia	0.50 ± 0.01a (0%)	0.51 ± 0.01a (0%)	0.50 ± 0.01a (8.3%)
*R. linaresii*	Italy, Siciliy: Trapani, foothills of Monte Cofano	0.58 ± 0.01c (0%)	0.60 ± 0.00a (20%)	0.55 ± 0.00b (80%)
*R. variicolor*	Italy, Sicily: Ragusa, Scicli	0.62 ± 0.01a (0%)	0.61 ± 0.01b (12%)	0.63 ± 0.01ab (38%)
	Malta: Gozo, Gharb	0.53 ± 0.01a (0%)	0.53 ± 0.01a (15%)	0.55 ± 0.01a (68%)
*R. ramiflora*	Italy, Tuscany: Isola d'Elba, Lacona	0.65 ± 0.01a (0%)	0.64 ± 0.01a (0%)	0.65 ± 0.01a (15%)
	France, Corsica: Farinole, Grotta u Banditu	0.52 ± 0.01a (0%)	0.52 ± 0.01a (0%)	0.51 ± 0.01a (13%)
	Italy, Sicily: Ragusa, Marina di Modica	0.57 ± 0.01a (0%)	0.57 ± 0.01a (0%)	0.56 ± 0.01a (33%)
*R. requienii*	France, Corsica: Ajaccio, Capo di Feno	0.59 ± 0.01a (0%)	0.59 ± 0.01a (0%)	0.60 ± 0.01a (1%)
	Italy, Sardinia: Olbia, Coda Cavallo	0.53 ± 0.01a (0%)	0.53 ± 0.01a (0%)	0.52 ± 0.01a (1%)
	Italy, Sardinia: Gesturi, Giara	0.62 ± 0.01a (0%)	0.63 ± 0.01a (0%)	0.63 ± 0.01a (0%)

Values followed by different letters within a column or different lowercase letters within a row are significantly different at the *P* < 0.05 level (Tukey's multiple comparisons test). The number in parentheses is the percentage of germination (radicle emergence).

**Table 3:** Results of the reduced binomial generalized linear mixed models (GLMMs) applied to seed germination data for each species. The following fixed factors (and interactions) were considered: mean temperature, alternating temperature, light, and warm stratification. The population was considered a random factor, as indicated

Fixed effects	Coefficient	SE	*z* value	*P*
*R. columnae*. Random effect: Population = 5, Variance 0.118, SD 0.343				
intercept	9.89	0.36	27.85	< 0.001
Mean temperature	−0.59	0.02	−29.72	< 0.001
Warm stratification	−3.57	0.30	−11.91	< 0.001
Alternating temperature	−2.24	0.13	−17.64	< 0.001
Mean temperature × Light	−0.14	0.01	−16.38	< 0.001
Warm stratification × Light	1.24	0.10	12.24	< 0.001
Mean temperature × Warm stratification	0.13	0.02	5.45	< 0.001
Warm stratification × Alternating temperature	3.32	0.15	22.71	< 0.001
*R. ramiflora*. Random effect: Population = 3, Variance 0.043, SD 0.208				
intercept	32.67	2.88	11.35	< 0.001
Mean temperature	−1.79	0.15	−12.15	< 0.001
Light	−2.87	0.78	−3.68	< 0.001
Alternating temperature	−2.80	0.81	−3.47	< 0.001
Warm stratification × Alternating temperature	2.24	0.41	5.51	< 0.001
Light × Alternating temperature	−3.14	0.88	−3.56	< 0.001
*R. ligustica*. Random effect: Population = 2, Variance 1.93, SD 1.389				
intercept	12.29	1.14	10.78	< 0.001
Mean temperature	−0.76	0.04	−21.41	< 0.001
Alternating temperature	−1.41	0.21	−6.81	< 0.001
Mean temperature × Warm stratification	0.04	0.02	2.92	< 0.01
Mean temperature × Light	−0.07	0.01	−6.46	< 0.001
Warm stratification × Alternating temperature	0.68	0.28	2.43	< 0.05
*R. variicolor*. Random effect: Population = 2, Variance 0.017, SD 0.001				
intercept	2.86	0.13	22.11	< 0.001
Mean temperature × Light	−0.08	0.01	−9.10	< 0.001
Mean temperature × Alternating temperature	−0.08	0.01	−9.44	< 0.001
*R. requienii*. Random effect: Population = 3, Variance 0.623, SD 0.789				
intercept	9.19	0.58	15.72	< 0.001
Mean temperature	−0.56	0.02	−23.76	< 0.001
Alternating temperature	−0.91	0.14	−6.25	< 0.001
Warm stratification × Alternating temperature	0.32	0.16	1.98	< 0.05
Warm stratification × Light	1.14	0.18	6.26	< 0.001
Mean temperature × Light	−0.08	0.01	−7.73	< 0.001

**Table 4:** Results of the reduced binomial generalized linear models (GLMs) applied to seed germination data for species with a single population. The following factors (and interaction) were considered: mean temperature, alternating temperature, light, and warm stratification

Effects	Coefficient	SE	*z* value	*P*
*R. insularis*				
intercept	22.62	2.06	10.98	< 0.001
Mean temperature	−1.67	0.14	−11.83	< 0.001
Warm stratification × Light	−1.41	0.54	−2.59	< 0.01
Mean temperature × Light	−0.09	0.04	−2.21	< 0.05
Mean temperature × Warm stratification	0.06	0.03	2.07	< 0.05
*R. linaresii*				
intercept	3.48	0.18	19.59	< 0.001
Mean temperature × Light	−0.11	0.01	−9.41	< 0.001
Mean temperature × Alternating temperature	−0.09	0.01	−8.26	< 0.001

Mean temperature was not a significant term for *R. linaresii* and *R. variicolor*. These two species germinated also at summer temperatures in the absence of light and especially under the constant temperature regime, and a significant negative interaction of mean temperature with light and with temperature regimes was detected. Sporadic germination under these conditions was also observed for *R. ligustica*,* R. ramiflora,* and *R. requienii* (Fig. 2). Next to mean temperature, the second most important factor that triggered germination was the temperature regime with alternating temperatures, which reduced the final germination percentage significantly.

The effect of light and warm stratification was dropped from most models, so that the next highly significant effects were the interactions between light and warm stratification and the interactions of these factors with the others. The interaction between the two factors light and mean temperature was significantly negative, which implies that the effect of light on germination depended on the mean temperature, with a marked negative effect at warm temperatures but no effect at cooler temperatures.

The absence of a main effect of warm stratification is in agreement with the fact that seed germination also occurred when seeds were directly placed under autumn or winter conditions, as reported above. However, although a warm stratification is not mandatory for the seed to germinate, it relaxes the requirements by widening the possible range of germination. This is shown by the highly significant positive effect of the interaction between warm stratification and other factors (Tables 3 and 4). Indeed, seeds of *R. ligustica* gained the ability to germinate at higher temperatures when moved from autumn to winter conditions, whereas seeds of *R. ramiflora* and *R. requienii* achieved better germination results under an alternating temperature regime or in the light. There was however one species, *R. columnae,* that particularly benefitted from a warm stratification (Fig. 2).

### Predictors of seed germination: relationship with incubation conditions, seed mass, and local climate

The phylogenetic logistic model showed that the main effects of mean temperature and the interactive effect of warm stratification with alternating temperature can predict seed germination (Table 5). Mean temperature had a strong negative effect, while the interaction between warm stratification and alternating temperature was a positive term in the model. Alternating temperature regimes were also an important explanatory variable, and the interactive effect of mean temperature and light predicted a lower probability of germination. Results were qualitatively similar, whether or not phylogeny was accounted for (Table 5).

**Table 5:** Results of the reduced phylogenetic (GEE) and across‐species logistic (GLM) models depicting the main effect of mean temperature, alternating temperature, light, and warm stratification for germination in the genus *Romulea*

Effects	Phylogenetic logistic model (GEE)				Across‐species logistic model (GLM)			
	Coefficient	SE	*t*	*P*	Coefficient	SE	*z* value	*P*
intercept	9.47	0.59	15.96	< 0.001	8.27	0.12	67.79	< 0.001
Mean temperature	−0.52	0.04	−13.37	< 0.01	−0.50	0.01	−62.80	< 0.001
Alternating temperature	−0.93	0.25	−3.74	< 0.05	−0.91	0.05	−17.82	< 0.001
Mean temperature × Light	−0.05	0.01	−3.74	< 0.05	−0.05	0.00	−17.55	< 0.001
Warm stratification × Alternating temperature	1.14	0.28	4.02	< 0.05	1.08	0.06	18.54	< 0.001

The germination response was also associated with seed mass and the local climate (Table 6). Looking at the bioclimatic variables (Table 1), our model simplification led us to retain the main effects of summer precipitation as a predictor of seed germination, with species from sites with higher summer precipitation having a stronger dormancy while species from wet habitats showing a weaker dormancy.

**Table 6:** Results of the reduced logistic model (GLM) depicting the main effect of habitat, climatic variables, and seed mass for germination in the genus *Romulea*

Effects	Coefficient	SE	*z* value	*P*
(Intercept)	−1.63	0.11	−14.7	< 0.001
Habitat moisture	0.26	0.02	9.80	< 0.001
Summer precipitation	−0.11	0.01	−9.36	< 0.001
Seed mass	4.94	0.18	26.75	< 0.001
Warm stratification	1.34	0.02	46.52	< 0.001

## Discussion

### Temperature range and the role of light: general considerations

*Romulea* species in the Mediterranean are characteristic for dry or temporarily humid grasslands where favourable conditions for the completion of the life cycle of a plant coincide with the rainy season during the coolest months of the year. Indeed, as most of the species germinated better at cool temperatures (< 15°C), this temperature range is consistent with their origin, and their ecology is typically Mediterranean (Doussi and Thanos 2002; Carta et al. 2013). Species studied produce a high number of water‐permeable seeds that germinate promptly after the summer season, which implies that they are able to rapidly colonize available space, which could be an advantage considering the high spatiotemporal variability of their habitats. However, it also implies a high level of intraspecific competition.

Variation among populations of the same species was low or even absent which might be explained by the fact that they share a similar climate and especially occur in the same habitat type. Variation in seed germination requirements and/or dormancy is generally detectable under stronger environmental gradients (Andersson and Milberg 1998; Carta et al. 2016).

Seed germination is not inhibited by darkness. Instead, seed germination is to some degree reduced in the presence of light, especially at warmer temperatures and/or under alternating temperature regimes. Both dark and constant temperature preferences for germination denote an advantageous strategy to avoid radicle emergence at the soil surface and thus decreases the likelihood of seedling mortality due to irregular inconstant water availability (Doussi and Thanos 2002; Fenner and Thompson 2005; Skourti and Thanos 2015).

The fact that overall germination was relatively rapid (10–20 days, see above) is in agreement with the absence of embryo growth. However, the phylogenetic regression revealed a weak embryo growth for the genus as a whole because the analysis also included data of the out‐group (*Crocus*), for which embryo growth was reported (Carta et al. 2014). Nevertheless, despite the fact that the embryo does not entirely fill the seed (as it is the case in many other seeds of plant species belonging to the family Iridaceae, see Martin 1946; Vandelook and Van Assche 2008; Carta et al. 2014; Skourti and Thanos 2015), embryo elongation in these *Romulea* species is not required for radicle emergence. A broader phylogenetic comparative study is required to understand the extent of this phenomenon in relation to the geographic origin and to the application of seed dormancy classification (Baskin and Baskin 2004). At this stage, we can say that *Romulea* seeds can likely be classified as nondormant regarding the morphological component. However, as warm stratification relaxes germination requirements by widening the possible range of germination, *Romulea* seeds should be classified as conditional‐dormant regarding the physiological component (Baskin and Baskin 2004).

### Timing of germination, germination requirements, and relation to local climate/environment/habitat

In general, both laboratory and outdoor experiments suggest a field germination timing that is synchronized with the rainy season in the Mediterranean. We found however ecologically meaningful differences between the species, suggesting the onset of adaptation to local ecological factors. Regarding germination phenology outdoors, two groups could be recognized: species starting to germinate when the temperatures reached 20–15°C (= early autumn; *R. linaresii, R. variicolor, R. ramiflora*) and those species starting to germinate when temperatures dropped to 15–10°C (= mid autumn; *R. columnae, R. insularis, R. ligustica*,* R. requienii*). Those species which germinated earlier in the outdoor experiment and in the laboratory are those which showed a broader germination window and which possessed heavier and bigger sized seeds; conclusions from previous attempts to relate seed mass to germination characteristics are not clear (Milberg et al. 1996; Fenner and Thompson 2005). Among the species studied here, those generally occurring in habitat types with lower water limitations and dry summers germinate readily when autumn rains commence. On the contrary, species from sites with higher summers precipitation and dry habitats show a higher degree of dormancy in order to avoid sporadic germination events under unfavorable ecological conditions. One species, *R. requienii*, is a typical species of Mediterranean temporary ponds (shallow water bodies which undergo periodic cycles of flooding and drought, Deil 2005). Although *R. requienii* grows in a habitat which does not show any water limitations during the autumn‐spring period, its germination timing and requirements are much more similar to those species which occur in dry pastures. We assume that with this strategy, seeds avoid germination during the first weeks of autumn rain, thus waiting for the temporary pool to be fully flooded later in autumn. Interestingly, our data also indicate that *R. requienii* does not show specific “aquatic” requirements (Probert 2000; Carta et al. 2013) for germination. Flooding in autumn deprives the seeds of oxygen, permitting germination to occur only in very shallow waters (10 cm) or at the pond margins. In cases of high temperatures (> 15°C), a secondary dormancy seemed imposed in *R. columnae* imbibed seeds. Indeed, under the constant temperature regime, fewer seeds which had been warm stratified germinated when moved to cooler temperatures, while the opposite effect was detected under the alternating temperature regimes. *R. columnae*, however, is the species that benefitted most from warm stratification. This is interesting because such a strategy is likely to be advantageous for this species, permitting germination only after the first autumn period characterized by warm temperatures. In addition, as reported for *Muscari* (Doussi and Thanos 2002), the delay in germination timing of *R. columnae* could be considered an ecological adaptation toward the Mediterranean climate with its often unpredictable rainfall pattern. However, the most delayed germination timing was observed for *R. insularis*, despite this plant growing in humid habitats. One likely explanation for this phenomenon lies in the fact that this narrow endemic species grows in sites which are exposed to strong winds where winter temperatures can be significantly cooler (5°C) than winter temperatures for the other species. To delay germination can be seen as a strategy of the species to avoid cold damage of the seedlings that might otherwise occur. On the contrary, instead of delaying germination itself, many Mediterranean montane species and those species from temperate climates show an epicotyl dormancy (Baskin and Baskin 2014) or a slow continuous seedling development after radicle emergence (Vandelook and Van Assche 2008; Carta et al. 2014) as alternative strategies to avoid cold damage. Nevertheless, shoot emergence immediately following radicle emergence in all studied *Romulea* species as in the Mediterranean harsh winter conditions is very rare.

### Seed germination response and phylogenetic relationship

The seed germination mechanism in *Romulea* seems to be adapted to habitat and climate conditions. Here, we can confirm our hypothesis that phylogenetically closely related plant species had similar ecophysiological traits (in our case, dormancy and germination behaviour). The main clade including *R. ramiflora* and the subclade with *R*. *variicolor* and *R. linaresii* are highly supported by sharing an early germination phenology and a preference for similar habitats. In addition, within the main clade, the subclade *R. variicolor* and *R. linaresii* is sharing very similar germination preferences under laboratory conditions. The second main clade composed of the remaining taxa includes those species which germinate later. Interestingly, the subclade *R. columnae*‐*R. insularis* is finely supported by our results, with seeds possessing stronger dormancy. The other subclade is less identifiable from a functional point of view, because the germination of *R. requienii* is more similar to *R. columnae* instead of being similar to *R. ligustica*.

Overall, however, considering the phylogenetic regression, the phenology of seed germination is more similar between the species than we expected by simply looking at the phylogenetic relationships between the species. One explanation is that the presence of multiple populations per species shows no differences.

As reported above, the germination requirements and the timing of the germination (radicle emergence) appear to be associated with an adaptation to the seasonal climate in the Mediterranean Basin. Cool temperatures and the tendency to germinate in the dark are also reported for southern African representatives of the genus *Romulea* (Swart et al. 2011). It is likely that these shared germination preferences are the result of convergent evolutionary processes, rather than being the result of phylogenetic constraints, as these patterns are reported for many geophytes growing in a Mediterranean climate (see, Doussi and Thanos 2002; Marques and Draper 2012). We do not know to what extent the observed phenology is the result of natural selection happening during species divergences, or whether phylogenetic constraint is more important in determining these patterns. However, we conclude that, while phylogenetically related species show very similar germination requirements, subtle but ecologically meaningful differences are present, confirming the onset of adaptation to local ecological factors mediated by species relatedness.

## Conflict of interest

The authors declare that they have no conflict of interest.
